# Death of Dr. E. M. Campbell, of Virginia

**Published:** 1878-07-20

**Authors:** 


### DEATH OF DR. E. M. CAMPBELL, OF VA.

At a meeting of the Abingdon Academy of Medicine, on the 13th ult., a committee appointed for the purpose presented the following eloquent tribute which was unanimously adopted:

"A melancholy duty has devolved upon your committee — that of noticing, in a formal manner, the death of one of our Fellows, Dr. Edward M. Campbell, of Abingdon, Virginia, who departed this life at the Warm Springs, North Carolina, on the night of the 11th inst.; and in discharing that duty we record, with no ordinary pleasure, the estimate we have of Dr. Campbell's exalted worth as a physician, surgeon, gentleman and citizen. His reputation as a physician and surgeon, is not confined to the particular locality in which he so long and so successfully practiced his profession, but extends throughout a large circle of the medical profession, who learned to regard him as a physician of great intelligence, of profound ability and a successful practitioner. As a citizen he stood very high in the estimation of all classes. He was a friend alike to the rich and the poor. By all his loss will be deplored. We submit the following resolutions:

1. *Resolved.* That in the death of Dr. Edward M. Campbell, this society has lost one of its brightest ornaments and most active members, a friend and gentleman, as well as an eminent physician and surgeon.

2. *Resolved.* That we not only deplore the loss that this society has sustained, but as citizens we regard the death of Dr. Campbell a great public loss.

3. *Resolved.* That the sincerest sympathies of the Abingdon Academy of Medicine be tendered the sorely afflicted family of Dr. Campbell; and, we do assure them we feel most deeply and pro foundly the heavy blow that has befallen them.

4. *Resolved.* That the editors of the "Abingdon Virginian," "The Standard," the "Virginia Medical Monthly," and the "Southern Medical Record' be respectfully requested to publish this paper, and that it be made of record upon the minute book of the Society."

Respectfully submitted,

W. F. Barr, M.D.,

J. S. Apperson, M.D., } Committee.

Geo. E. Wiley, M.D.

Abingdon, Va., June 17, 1878.

